# Robust encoding of stimulus–response mapping by neurons in visual cortex

**DOI:** 10.1073/pnas.2408079122

**Published:** 2025-02-24

**Authors:** Donatas Jonikaitis, Ruobing Xia, Tirin Moore

**Affiliations:** ^a^HHMI, Stanford University School of Medicine, Stanford, CA 94305; ^b^Department of Neurobiology, Stanford University School of Medicine, Stanford, CA 94305

**Keywords:** memory, cognition, sensorimotor integration, visuomotor, affordance

## Abstract

Neural activity in sensory cortex is modulated by behavioral and cognitive factors, and this modulation is thought to contribute to the selection of specific sensory information needed to achieve behavioral goals. In contrast, more abstract behavioral variables that are independent of stimulus selection, such as stimulus–response mapping, are thought to be encoded by neurons outside of sensory cortex. We show that information about such mapping is robustly encoded in the responses of neurons in primate visual cortex. Monkeys were trained to alternate between two tasks that differed in the rule governing the mapping of a remembered visual cue onto an eye movement response. During the memory-delay period, neurons in area V4 reliably signaled the remembered cue location in both tasks. However, the encoding of cue location depended critically on the stimulus–response mapping rule. Thus, V4 delay activity encoded the mapping rule and signaled the preparation of the appropriate motor response rather than spatial working memory per se, contrary to previous assumptions. In addition, we probed the origins of motor-related delay activity and found that it was reduced during local inactivation of the frontal eye field (FEF). The results demonstrate that behavioral modulation of visual cortical activity is not solely related to the selection of sensory stimuli but instead reflects a distinct mechanism for sensory-guided motor output.

## Significance

Behavioral modulation of neural activity in sensory cortex is thought to contribute to the selection of sensory information, rather than the representation of abstract behavioral variables. We show that during spatial working memory, neurons in primate visual cortex signal the mapping of stimuli onto motor responses, rather than the sensory attributes of memoranda. Monkeys performed two tasks differing in the rule governing the mapping of a remembered cue onto an eye movement response. Rather than signaling the remembered cue location independent of the task rule, memory delay activity of area V4 neurons signaled the appropriate motor response. Consistent with a motor influence on V4 delay activity, that activity was reduced during local inactivation of the frontal eye field (FEF).

Many behaviors can rely solely on fixed stimulus–response associations. However, more complex behaviors, particularly those of primates, rely on the ability to flexibly assign different behavioral responses to the same stimuli, depending on the context. For example, orienting toward a stimulus under one rule, yet avoiding the stimulus under a different rule requires flexible command of behavioral output, and it is a hallmark of executive control (1). Neural activity in visual cortex is modulated by behavioral factors such as attention (2, 3), reward value (4), and working memory (5, 6), and in such cases, sensory responses reflect a selection of the specific sensory information needed to achieve behavioral goals. In contrast, more abstract behavioral constraints that do not involve stimulus selection, such as stimulus–response mapping rules, are thought to be encoded by neurons outside of sensory cortex (7). The neural mechanisms of behavioral flexibility have been studied extensively in both human and nonhuman primate models, where a dominant role of prefrontal cortex in the implementation of abstract rules is well established (7, 8). For example, neurons in prefrontal cortex exhibit robust representations of the abstract rules governing the mapping of sensory stimuli onto appropriate behavioral responses (9). These representations appear to coexist with movement-related signals involved in controlling behavioral responses, such as eye movements, though they appear to be dissociable from them (10, 11). In contrast, neurons in posterior sensory areas are not generally considered as having an active role in movement control, and thus the extent to which they convey signals about stimulus–response mapping remains an open question.

Behavioral modulation of visual cortical activity results in the selection of specific information by visually responsive neurons, including information about stimulus features (4, 12) or location (5, 13–15). This modulation is generally thought to contribute to changes in stimulus-related aspects of behavioral performance, such as perceptual sensitivity to attended stimuli (2, 3), reinforcement of particular features (4, 16), or memory of relevant locations (5, 6, 17). In such cases, the selection of stimulus information is inherent in each behavioral condition, and modulation is observed when some spatial, featural, or object-related component of a visual stimulus is rendered relevant, typically via some form of a cue. Thus, the involvement of visual neurons in signaling selected stimulus dimensions seems consistent with their role in visual perception. Much less clear is whether information about stimulus–response mapping rules is also represented in sensory representations. Often, the rule governing performance on a particular task can be abstract, and thus orthogonal to any particular dimension of sensory input and may merely identify appropriate mappings between sensory input and behavioral responses (1, 8).

Modulation of neural activity during the delay period of working-memory tasks has been observed throughout the brain of human and nonhuman primates (18–23). Among the models that have emerged from this evidence is the notion that working-memory-related signals across the brain reflect a gradient of abstraction from sensory areas, where low-level sensory features are represented, to parietal and prefrontal regions, where more abstract, and motor-related signals dominate (23). For example, one study found that, in comparison to premotor and prefrontal cortex where a high proportion of neurons signaled task rules, only very few neurons signaled task rules within inferotemporal (IT) cortex, the final stage of the ventral visual system (24). As in IT cortex, neurons in earlier visual cortical areas exhibit modulated activity in the absence of visual stimulation while spatial or featural information is held in working memory (5, 13, 21, 25–27). Accordingly, such modulation in visual cortex is generally thought to contribute to the temporary storage of sensory details (e.g., retinotopic location), whereas the more abstract and movement-related information is thought to be represented in parietal and frontal cortex (23). Consistent with this notion, working memory-related activity in area V1 was found to appear independent of the stimulus–response map (6). However, this assumption has not been adequately tested.

We measured the extent to which neurons in primate area V4 represented a task rule, specifically a stimulus–response mapping rule. Monkeys were trained to alternate between two tasks that differed in the rule governing the mapping of a remembered visual cue onto an eye movement response. We assessed whether neuronal activity present during the memory delay period signaled the remembered cue location, and whether that signal was dependent on, or independent of, the stimulus–response mapping rule. Next, we tested the dependence of memory delay activity on input from the frontal eye field (FEF), the primary source of prefrontal input to posterior visual cortex.

## Results

### Behavioral Response Preparation in the Look and Avoid Tasks.

**Fig. 1.**
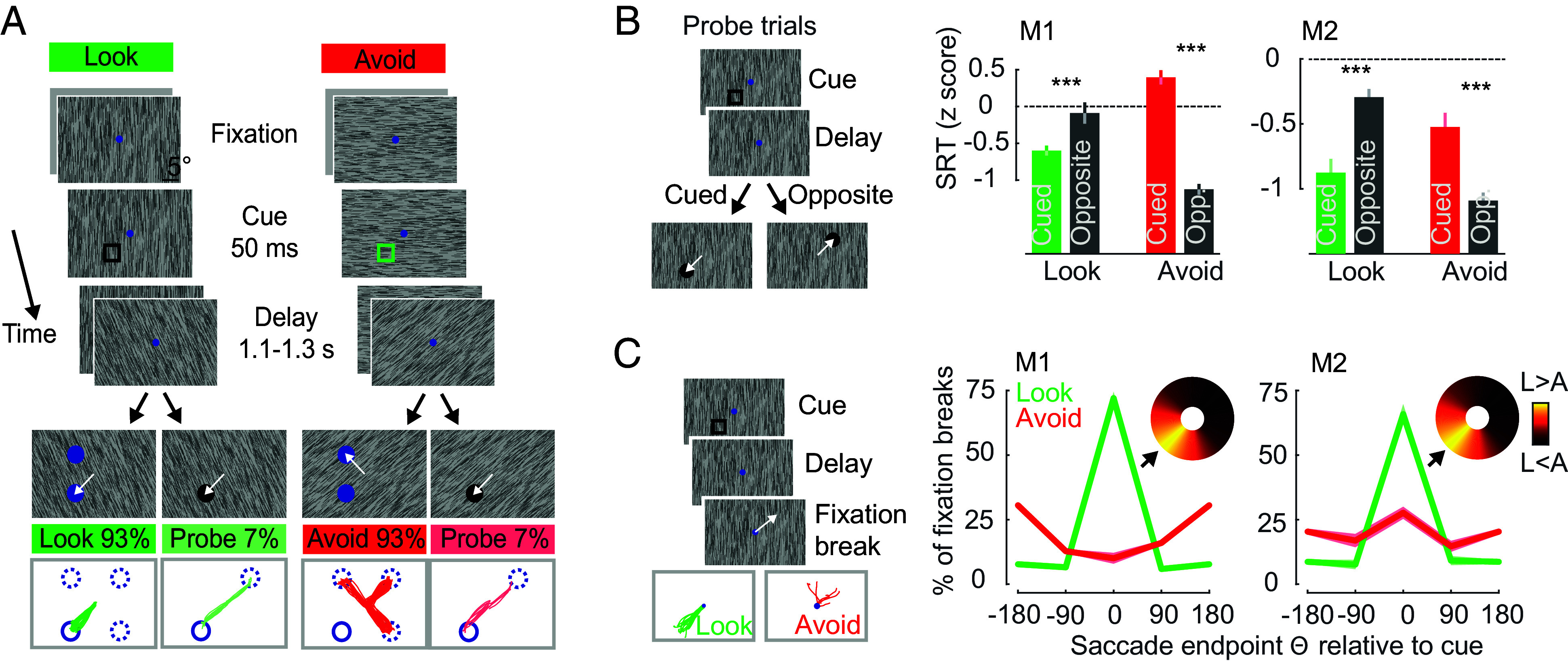
Task paradigms and behavioral results. (*A*) In both tasks, following central fixation, a briefly presented cue (open square) indicated the location to be memorized. On the majority of trials, after a delay period, two response targets appeared, one at the cued location and one at a location randomly selected from the noncued locations. In the Look task, monkeys made a saccadic eye movement to the target matching the cued location. In the Avoid task, monkeys made an eye movement to the target at the nonmatching location. The cue color differed between the different tasks to highlight the current rule. On a small proportion of trials, only one response target (probe) appeared, and the monkey was rewarded for eye movements to the probe. The probe was presented at either the cued or the opposite location. On most trials, the display contained a task-irrelevant background texture; on the remainder of trials, it was uniform gray. *Bottom* row: Example eye movement responses relative to the cue location during task and probe (monkey AQ). Response targets at cued locations are shown as solid circles, and at noncued locations as dashed line circles. (*B*) Saccadic reaction times (SRTs) measured during probe trials. Probes appeared either at the cued location, or the location opposite from the cue (*Left*). Z-score normalized SRTs on probe trials, for monkeys M1 and M2. Significant statistical comparisons (*P* < 0.001) between the cued and opposite locations are denoted with ***. (*C*) Illustration of fixation breaks during the memory delay (*Left*). *Bottom* panels show example eye movement traces during fixation breaks. Eye traces are rotated so that the cue location is at the *Lower Left*. *Right*, percentages of fixation breaks during the memory delay relative to the cue location for M1 and M2. Heatmaps in *Upper Right* depict differences in fixation breaks between Look and Avoid trials (L–A). Data are rotated so that the cue location is at the *Lower Left* (arrow).

We trained two monkeys to perform two versions of a task in which they either looked at or avoided looking at a memorized location (Fig. 1*A*) (10, 28, 29). In the “Look” task, monkeys memorized a cue location and after a delay period, were rewarded for making an eye movement to a target (match) appearing at the cued location. In the “Avoid” task, monkeys were instead rewarded for making an eye movement to a target appearing at a noncued location (nonmatch). Although the behavioral responses differed between the two tasks, looking at or avoiding the cued location, neither could be solved without memorizing that location. Monkeys performed alternating blocks of Look and Avoid trials (median = 218 trials/block; range = 75 to 438 trials) during each experimental session (monkey AQ: 41 sessions; monkey HB: 18 sessions). Both monkeys performed the two tasks successfully (median performance AQ: Look = 94%, Avoid = 88%, *P <* 0.001; HB: Look = 75%, Avoid = 72%, *P =* 0.18), and performance was similar to studies using multitask designs, such as prosaccades and antisaccades in the same experimental session (30–32).

Although both tasks required memorizing the cue location, only in the Look task was the eventual eye movement response known to the monkey during the memory delay. Thus, the Look task permitted the preparation of movement responses to the memorized location, whereas the Avoid task did not, and instead designated a location to which a movement could not be made (10, 29). We assessed response preparation during both tasks. On a small number of trials (7%), we presented a single response target (probe) and monkeys were rewarded for making a saccadic eye movement to it, regardless of whether the probe was at the cued or noncued location (Fig. 1*B*). Saccadic reaction times (RTs) are generally faster to probes at cued versus noncued locations, consistent with covert orienting and response preparation (33–36). Indeed, we also observed faster RTs to probes at cued locations versus noncued locations in the Look task (AQ: *P* < 0.001, HB: *P* < 0.001). In contrast, RTs to probes at cued locations were slower in the Avoid task (AQ: *P* < 0.001, HB: *P* = 0.003). Thus, response preparation toward and away from the cued location clearly differed in the two tasks.

On some trials, monkeys failed to maintain fixation during the memory period and made saccades to other locations, which aborted the trial and provided no reward. These fixation breaks can reveal spatial biases in response preparation during Look and Avoid tasks (37) (Fig. 1*C*). We observed that during the memory period (−300 to 0 ms before delay end), the directions of fixation breaks were strongly influenced by the task. The proportion of fixation breaks toward the cue was much higher in the Look task than in the Avoid task (Look-Avoid difference: AQ 75 ± 5.3%, *P* < 0.001; HB 36.3 ± 4%, *P* < 0.001). In contrast, the proportion of fixation breaks away from cue was higher in the Avoid task (AQ: −40.1 ± 4.7%, *P* < 0.001; HB −15.8 ± 2.2%, *P* < 0.001). Moreover, unlike in the Look task, there was no consistent bias in the direction of fixation breaks in the Avoid task. Thus, during the memory period of the Look task, monkeys appeared to be preparing saccades to the cued location. When combined, both the probe trials and the fixation break trials provide evidence that the preparation of eye movement responses differed dramatically between the Look and Avoid tasks. In short, as might be expected, cues in the Look task were followed by a bias in the preparation of movements to the memorized location. In contrast, during the Avoid task, cues were followed by a relative bias in the preparation of movements to other locations.

### Dependence of V4 Delay Activity on Stimulus–Response Mapping.

The Look and Avoid tasks both required monkeys to memorize the location of the cue. However, the behavioral results indicate that the preparation of the behavioral response differed between the two tasks. Previous studies argue that memory-related activity in sensory areas does not encode information about task rules or motor preparation (5, 6, 23). However, this assumption has not been thoroughly tested. Thus, we asked whether neurons in visual cortex encode information about remembered cues, independent of a stimulus–response mapping rule. Specifically, we asked whether the pattern of memory delay activity in area V4 was the same or different between the Look and Avoid tasks. Area V4 is a midlevel visual area containing neurons highly sensitive to shape, texture, and color (38–41). Neuronal activity in V4 is also known to be modulated during tasks involving attention (42–44), reward expectation (4), and working memory (13, 25).

**Fig. 2.**
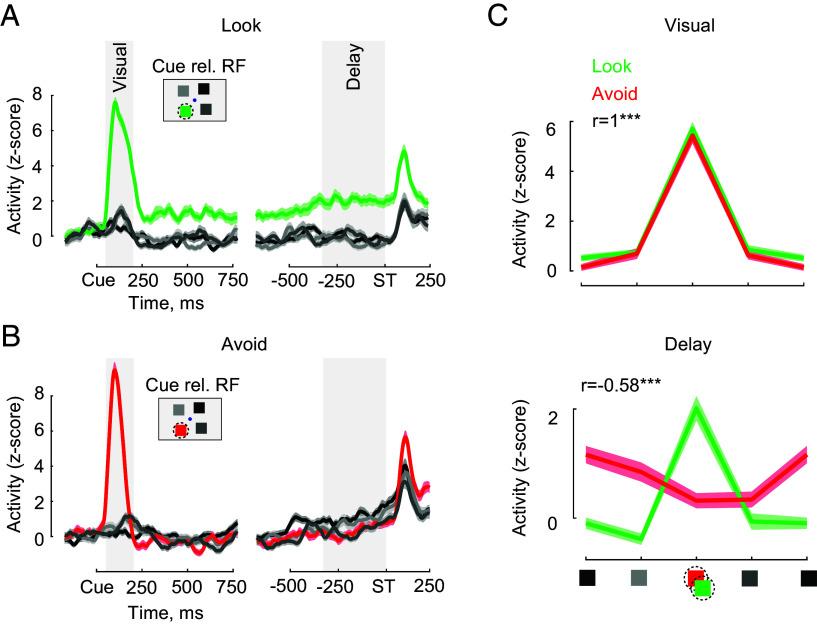
Example V4 delay activity. (*A*) Example neuronal activity in the Look task. Activity is shown relative to cue onset time (“Cue”) and relative to delay end and saccade target onset (“ST”). The inset shows cue locations on screen, and RF location (dashed circle). Shaded areas show visual and delay period intervals used for analyses. (*B*) Example neuronal activity in the Avoid task. (*C*) Mean neuronal activity during the visual and delay periods in the Look and Avoid tasks (same unit as in *A* and *B*). Cue locations relative to RF location (dashed circle) are shown with the same colors as panels *A* and *B*. Data from the cue-Opposite location are plotted twice to visualize the full tuning cycle. Correlation (r) of activity between Look and Avoid tasks is shown in each epoch. ***P < 0.001.

Neuronal activity in V4 was recorded with linear array electrodes that provided simultaneous single and multiunit recordings at 16 to 32 sites distributed across the cortical depth. During each task, one of the cue locations was positioned within the receptive field (RF) of recorded neurons. Single and multiunit activity was combined totaling 1,442 units across 57 sessions in the two monkeys (AQ: 39 sessions, 982 units; HB: 18 sessions, 460 units) (*Materials and Methods*). During the Look task, neurons in V4 responded initially to the visual cue when it appeared within the RF. Following the removal of the cue, while the animal remembered its location, many neurons exhibited elevated activity throughout the delay period (Fig. 2*A*) similar to neurons in parietal (45) and prefrontal cortex (7, 19, 46, 47) and consistent with previous studies in V4 (13). At the end of the trial, neurons responded again to the matching target appearing in the RF. In contrast, during trials in which the cue appeared outside of the RF, activity was often suppressed below baseline levels during the memory delay period. At the end of the trial, neurons responded to the nonmatching target appearing in the RF.

The responses of V4 neurons during the Avoid task differed dramatically from that of the Look task (Fig. 2*B*). Neurons responded initially to the visual cue appearing in the RF and to the matching targets at the end of the trial, like the Look task. However, during the delay period, neurons failed to exhibit elevated activity. Instead, in this condition, activity was equal to or lower than that of the non-RF cue conditions. In the example, the spatial tuning of activity across cue locations was highly correlated between the two tasks during the initial cue (visual) period (r = 1.0, *P* < 0.001) (Fig. 2*C*). However, the tuning of memory delay activity was significantly anticorrelated between the two tasks (r = −0.58, *P* < 0.001).

**Fig. 3.**
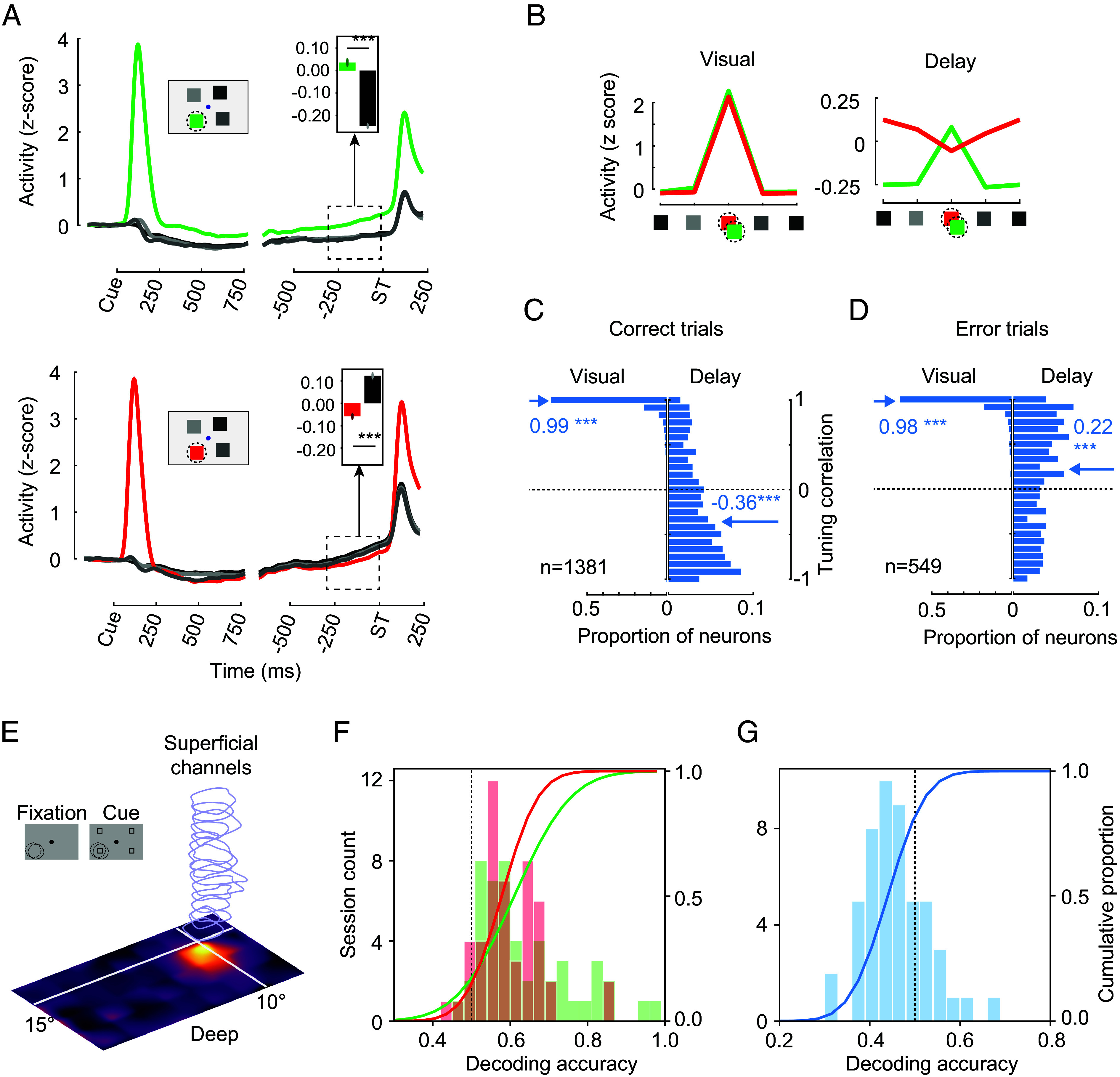
Population delay activity and decoding of remembered cue location in single recording sessions. (*A*) Normalized mean activity of all V4 neurons with visual RFs during the Look (*Top*) and Avoid (*Bottom*) tasks. Insets compare mean activity between cue-RF and cue-Opposite locations at the end of the delay period. Note that differences in activity following ST onset reflect differences in the probability of stimuli appearing within the RF (cue-in: 100%, cue-out: 33%). (*B*) Mean neuronal activity during the visual and delay periods in the Look and Avoid tasks. (*C*) Distributions of correlations between Look and Avoid activity during the visual (*Left*) and delay (*Right*) periods across the population of V4 neurons on correct trials. (*D*) Distributions of correlations between Look and Avoid activity during the visual (*Left*) and delay (*Right*) periods across the population of V4 neurons on error trials. Arrows indicate median values. Mean correlation coefficients are shown in blue. Other conventions as in previous figures. (*E*) An example linear array recording showing neuronal RFs measured across 16 recording sites spaced every 150 µm across the cortical depth (2,250 µm total). (*F*) Distributions of decoding accuracy across sessions during the Look (green) and Avoid (red) tasks. Lines show cumulative functions. (*G*) Distribution of accuracies of decoders trained on Look trials and tested on Avoid trials across sessions.

We observed the above pattern of results across the full population of V4 neurons (n = 1,442) in the two monkeys. During the Look task, neurons responded initially to the visual cue appearing in the RF. Subsequently, during the memory delay, activity was elevated in comparison to the non-RF cue conditions. In the Avoid task, that pattern was reversed. After the initial response to the RF cue, neuronal activity remained at or below the level of the non-RF cue conditions. We assessed the selectivity of neurons to the remembered cue location in the two tasks during the last 300 ms of the delay period, just prior to the appearance of the match/nonmatch targets. Specifically, we compared the delay activity in the cue-RF condition to that of the cue-Opposite condition (delay selectivity) (Fig. 3*A*). Across the overall population of neurons, delay selectivity differed significantly from 0; it was positive for Look and negatively for Avoid (Look, mean delay selectivity = 0.31; *P* <0.001; Avoid, mean delay selectivity = −0.18; *P* < 0.001). This effect was significant in both monkeys (AQ: Look and Avoid *P* < 0.001; HB: Look and Avoid, *P* <0.001). Single and multiunit activity also exhibited individually significant effects of cue location in the delay period (AQ: 43.1%, Look, 31.3%, Avoid; HB: 19.1%, Look, 7.2%, Avoid). Thus, V4 neurons were selective to the remembered location in both tasks. Yet, the pattern of selectivity differed between them. Specifically, we observed greater activity in the cue-RF condition during the Look task, and lower activity in the Avoid task. As in the example, the spatial tuning of activity across all cue locations was highly correlated between the two tasks during the initial visual response to the cue. However, that relationship changed dramatically during the delay period (Fig. 3*B*). During the visual period, the mean spatial tuning correlation between the Look and Avoid tasks was 0.99 (*P* < 0.001) (Fig. 3*C*). In contrast, during the delay period, the mean tuning correlation between the two tasks became significantly anticorrelated (r = −0.36, *P* < 0.001). We confirmed that these effects were independent of microsaccades (*SI Appendix*, Fig. S1) and that they were not a result of blocked Look and Avoid trials (*SI Appendix*, Fig. S2 *A*–*C*). Rather, differences in the pattern of delay activity between the two tasks resulted from differences in the stimulus–response mapping rule.

Although performance in both tasks was relatively high, monkeys occasionally made errors, responding to the nonmatching target in the Look task and the matching target in the Avoid task (AQ: Look, 9%, Avoid, 14.7%; HB: Look, 23.3%, Avoid, 27.3%) (*Materials and Methods*), the latter being more frequent. Thus, on these trials, the behavioral responses were consistent with a neglect of the stimulus–response mapping rule, more often in the Avoid task. Thus, we considered that activity in the delay period might reflect such errors in the mapping rule. Indeed, we observed that the correlation in delay tuning between the two tasks became significantly positive on error trials (r = 0.22, *P* < 0.001) (Fig. 3*D*), an effect that differed significantly from that of correct trials (*P* < 0.001). This result is consistent with error trials being associated with an incorrect encoding of the stimulus–response rule during the delay period.

Our measurement of neuronal activity from simultaneous recordings across the cortical depth of V4 (Fig. 3*E*) allowed us to assess the degree to which local populations of neurons (15 to 32 units) encoded the remembered cue. Furthermore, it allowed us to compare the encoding of cues across the two tasks (AQ: 32 sessions; HB: 18 sessions). We first trained a binary (location) decoder on the delay activity from these simultaneously recorded populations during each task and measured its accuracy in distinguishing between cue-RF and cue-Opposite trials using a leave-one-out cross-validation scheme (*Materials and Methods*). During both the Look and Avoid tasks, mean decoder performance exceeded that of chance (Look: *P* < 0.001; Avoid: *P* < 0.001) (Fig. 3*F*). Location decoder performance was slightly greater during Look trials than during Avoid trials (Look: median = 59.4%, Avoid: median = 57.7%, *P* = 0.014, paired comparison), consistent with the differences in behavioral performance observed between the two tasks in both monkeys. Second, differences in spatial tuning evident in the individual neurons between the two tasks suggest that the stimulus–response mapping rule was encoded in the delay activity. If indeed true, then decoders trained on one task should fail to generalize to the other task and should mislocalize cue locations across tasks. Indeed, this is what we observed. Location decoders trained on the Look task and tested on Avoid trials on average performed significantly poorer than chance (median = 45.1%, *P* < 0.001) (Fig. 3*G*), incorrectly localizing the cue location during the memory delay period. Like the tuning correlation analysis, to control for effects of the block design, we compared the performance of decoders trained across tasks with those trained within each task but across similar periods of time. This comparison confirmed that the mislocalization was confined to decoders trained across tasks (*SI Appendix*, Fig. S2 *D*–*F*). Thus, the combined single-neuron and decoding results indicate that the stimulus–response mapping rule was encoded in the delay activity.

### Dependence of Memory Delay Activity on the FEF.

Our comparisons of activity between the Look and Avoid tasks indicate that rather than encoding information solely about the remembered cue location, V4 neurons signaled the preparation of the appropriate mapping of the eye movement response. If true, this predicts that delay activity in V4 should depend primarily on input from neurons more directly involved in eye movement control. Areas within primate extrastriate cortex, including V4, receive direct input from the FEF (48, 49), an area directly involved in the programming and triggering of saccadic eye movements (50–52). In addition, previous studies have established that FEF neurons are a source of modulatory input to visual cortex during for attention (53–56) and working memory (27). Thus, we considered that the FEF might contribute to the delay period selectivity of V4 neurons.

**Fig. 4.**
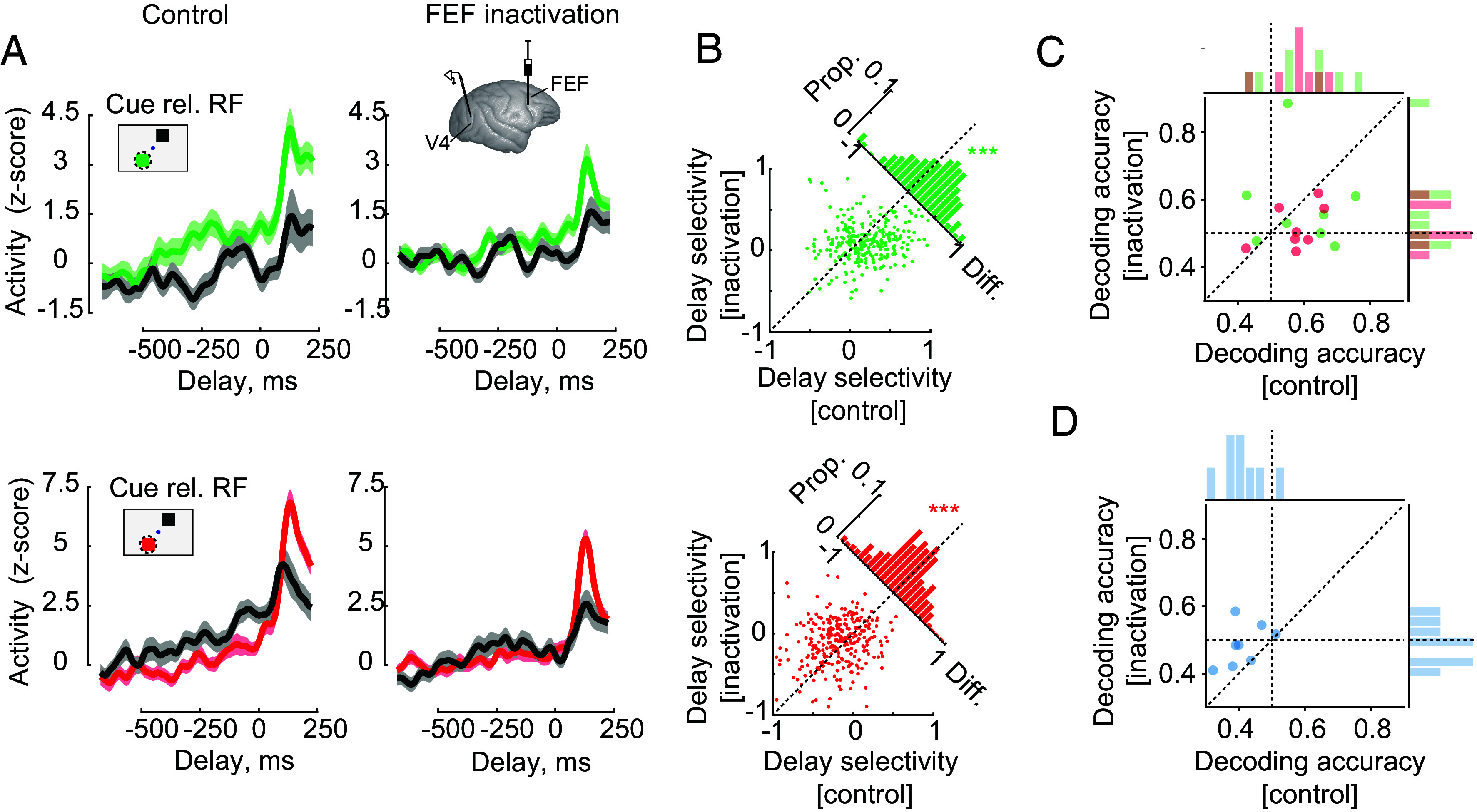
Changes in V4 delay activity and delay period decoding following FEF inactivation. (*A*) Recordings from V4 were conducted before and after local inactivation of the FEF at retinotopically overlapping sites. Example V4 recording before (*Left*) and after (*Right*) FEF inactivation during the Look (*Top*) and Avoid (*Bottom*) tasks. Neuronal activity for cue-RF and cue-Opposite conditions is shown. (*B*) Scatter plots compare delay selectivity (Cue-RF—Cue-Opposite) for control and inactivation across the population of V4 neurons. Diagonal histograms plot the distributions of changes in selectivity after inactivation. (*C*) Comparisons of population decoding of cue location during the delay period across sessions before and after inactivation during Look and Avoid trials. Marginal distributions show values relative to chance performance. (*D*) Comparison of the accuracy of decoders trained on Look trials and tested on Avoid trials before and after inactivation.

To test that, we carried out an additional set of V4 recordings (AQ: 7 sessions; HB: 3 sessions) both before and after pharmacological inactivation of the FEF (*Materials and Methods*) (Fig. 4). FEF inactivation was achieved via local injections of muscimol within discrete sites that overlapped retinotopically with RFs of the recorded V4 neurons, as in previous studies (57, 58). As expected from past evidence (28, 58, 59), inactivation of the FEF resulted in clear deficits in the performance of a memory-guided saccade (MGS) task (*SI Appendix*, Fig. S3). Inactivation increased both the reaction time of memory-guided saccades and the number of response errors. Deficits in the latter were evident across experimental sessions in the two monkeys (AQ: −12.2%; HB: −27.1%; combined, −19.5%, *P* < 0.001). As recently reported, FEF inactivation produces impairments that differ between the Look and Avoid tasks and are most consistent with deficits in the preparation of eye movements rather than working memory per se (28).

We measured the effects of the FEF inactivation on V4 memory delay activity during the Look and Avoid tasks. In 9 of the 10 combined inactivation-recording sessions in the two monkeys, there were sufficient trials completed during the Look and Avoid tasks to measure V4 activity both before and after inactivation (*Materials and Methods*). We measured the effects of FEF inactivation on V4 delay activity in a total of 280 single and multiunits (AQ, 184; HB, 96). We focused our analyses on correct trials to facilitate comparisons between control and inactivation conditions. We found that FEF inactivation reduced the effect of cue location on delay period activity in both Look and Avoid tasks (Fig. 4*A*). Specifically, the difference in activity between cue-RF and cue-Opposite conditions was reduced compared to control trials. Consequently, delay period selectivity was reduced across the population of neurons in both the Look and Avoid tasks (Fig. 4*B*). During the Look task, FEF inactivation reduced delay period selectivity by 31% (control—inactivation = 0.05, *P* < 0.001) and during the Avoid task inactivation reduced delay period selectivity by 50% (control—inactivation = −0.09, *P* < 0.001). Thus, delay period modulation in V4 depended on FEF activity.

As in the initial experiments (Fig. 3*F*), we also examined the degree to which local populations of neurons encoded the remembered cue in each recording session and the effects of the FEF inactivation. For each block of the Look and Avoid task with a minimum number of trials during both control and inactivation conditions, we trained a decoder on the delay activity from simultaneously recorded neurons (29 to 32 units) and measured decoding accuracy (*Materials and Methods*) (AQ: 5 sessions; HB: 3 sessions) (Fig. 4*C*). In spite of the relatively small number of total sessions, mean decoder performance was significantly above chance during both the Look and Avoid tasks prior to inactivation (Look: median = 60.05%, *P* = 0.007; Avoid: median = 57.68%, *P* = 0.001). However, following FEF inactivation, the decoder performance no longer differed significantly from chance in the Avoid task (median = 49.31%, *P* = 0.202; difference from control, *P* < 0.04), though it remained above chance in the Look task (median = 54.30%, *P* = 0.011; difference from control, *P* < 0.42). When data from the two tasks were combined, median performance during control trials was 57.7%, and was 51.7% following inactivation, a difference that approached significance (*P* = 0.09). In addition, during control trials, as in the initial experiments (Fig. 3*G*), decoders trained on the Look task and tested on the Avoid task on average performed significantly poorer than chance (median = 39.5%, *P* < 0.001) (Fig. 4*D*), incorrectly localizing the cue location during the memory delay period. However, following FEF inactivation, this effect was eliminated (median = 48.5%, *P* = 0.245), and performance differed significantly from that of control trials (*P* < 0.004). This effect appeared to result from a reduction in the encoding of cue location during the delay in either task, a reduction in the difference in the encoding of stimulus–response mapping between the two tasks, or both.

## Discussion

Our results demonstrate that V4 neurons robustly signal information about the mapping of visual stimuli onto specific motor responses. During a memory delay period, the activity of V4 neurons varied systematically with the location of remembered cues. However, the tuning of delay period activity differed dramatically between two tasks with different stimulus–response mapping rules. Specifically, during a task in which the remembered cue location was identical to that of an eye movement response, delay period activity was at its peak for cue locations within the neuronal RF. In contrast, during a task in which the movement response was unknown, but was guaranteed not to coincide with the remembered cue location, delay period activity was lowest for cue locations within the neuronal RF. Consequently, V4 neurons reliably signaled the stimulus–response mapping rule, rather than a rule-independent memory of cue location. Furthermore, decoding of V4 delay activity during individual recording sessions confirmed that the encoding of cue location was evident during the delay period in both tasks, but that it did not generalize across the two tasks. Finally, we probed the origins of motor-related delay activity and found that it was reduced during localized inactivation of the FEF. Together, the results demonstrate the robust influence that motor signals impose on the activity of visual cortical neurons even in the absence of visual input. These results run contrary to the view that modulation of visual cortical activity during working memory contributes primarily to the temporary storage of sensory details, rather than action-related information. Furthermore, they appear to contradict the notion that visual cortical areas primarily encode the sensory properties of remembered stimuli (6) rather than the “action features” of memoranda, similar to that expected of frontal and parietal cortical areas (23). Instead, they appear more consistent with the breadth of evidence demonstrating a potent influence of motor-related signals on sensory cortex (60–64).

It is well established that neuronal activity within area V4, and across many levels of the primate visual system, is modulated by behavioral and cognitive factors including attentional deployment, reward expectation, and working memory (2–6), and that V4 activity is not solely determined by the retinal stimulus. These forms of extraretinal modulation have generally been thought to contribute to changes in stimulus-related aspects of behavioral performance, e.g., improvement in perceptual performance during attention (65). Invariably, modulation by those behavioral and cognitive factors requires that some spatial, feature, or object-related component of a visual stimulus is rendered relevant, or given some lesser or greater reward value, typically via some form of a cue. Indeed, such experiments are specifically designed to measure the effects of cueing some stimulus dimension or another on neuronal activity. Notably, these cueing effects can be observed even in the absence of visual stimulation (6, 13, 15), and could reflect some form of prospective or retrospective selection of stimulus attributes (e.g., location).

In contrast, the rules governing behavioral performance on a particular task can be rendered orthogonal to any dimension of sensory input. For example, such rules may merely identify the appropriate mappings between sensory input and behavioral responses (1, 8). In contrast to prefrontal or parietal cortex, where neurons appear to signal a multitude of arbitrary rules and stimulus–response mappings (1, 8), one would not expect the same of visual cortical neurons, given the common view that visual cortical neurons function largely as perceptual filters, particularly areas within the ventral visual stream (66), such as area V4. However, our results indicate that V4 activity signals information about the stimulus–response rule governing task performance, independent of the perceptual and cognitive demands of the task. Were this not the case, we would expect that delay-period activity encoding the cue location would have done so independent of the motor response required, as observed in a previous study in V1 (6). Such a result would have been consistent with the notion that working-memory-related signals in sensory areas represent low-level sensory features, whereas more parietal and prefrontal areas represent more abstract, motor-related signals (23). However, at least at the level of area V4, a clear influence of motor-related signals is already evident.

Notably, we show that evidence of a motor influence on V4 activity is supported by a reduction in delay-period selectivity following local inactivation of the FEF, which is the part of dorsolateral prefrontal cortex with direct projections to much of retinotopic visual cortex (48) and the part most directly involved in the control of saccadic eye movements (50–52). Also consistent with this observation is the finding that FEF inputs to visual cortex appear to originate disproportionately from neurons with elevated delay-period activity (27) and from neurons expressing dopamine D1 receptors (67), which are implicated in the maintenance of persistent, delay-period activity (19, 68). More broadly, spatially selective delay-period activity in dorsolateral prefrontal cortex is thought to contribute to spatial working memory (69), and in some areas (e.g., area 46), delay-period activity of many neurons appears to encode remembered locations independent of motor plans (e.g., 19). Were such signals to be transmitted directly to retinotopic visual cortex, we might expect a similar pattern of results there. Instead, delay activity within V4 appears to recapitulate the properties of its direct inputs from the FEF, where neurons also encode planned movements rather than remembered locations when animals perform the same tasks that dissociate the two (70). Furthermore, consistent with the present results, a recent study shows that following inactivation of the FEF, the resulting behavioral impairments appear principally related to motor preparation rather than spatial working memory per se (28).

How generally might V4 neurons encode task rules? Although the two response mapping rules we employed were sufficient to yield clear differences in the pattern of delay-period activity, it need not follow that V4 neurons signal differences between any arbitrary set of rules. Rather, one might expect rule-related signals to emerge only in tasks that draw upon the unique functions of visual cortical circuitry, including perhaps nonperceptual functions. Evidence across species of a more active role of visual cortical neurons in coordinating gaze behavior has been accumulating for some time (60, 61, 71), yet such a role may not extend to other behaviors. The observation that V4 neurons signal information about how visual stimuli are to be mapped onto gaze shifts might reflect an involvement of this area, and perhaps other ventral visual areas, in the preparation of saccadic eye movements. However, this may not be the case for other types of visually guided behaviors, such as reaching and grasping. Future work will be needed to test the influence of other motor effectors on visual cortical activity, as has been explored in parietal and frontal cortical areas (45, 72). Nonetheless, the current results demonstrate that behavioral modulation of visual cortical activity is not solely related to the selection of sensory stimuli, but instead reflects a distinct mechanism for sensory-guided motor output.

## Materials and Methods

### General and Surgical Procedures.

Two male rhesus monkeys (Macaca mulatta, 10 and 13 kg), monkey AQ and monkey HB, were used in this study. All experimental procedures were in accordance with NIH Guide for the Care and Use of Laboratory Animals, the Society for Neuroscience Guidelines and Policies, and Stanford University Animal Care and Use Committee and are detailed in a previous report (47).

### Behavior Tasks: RF Mapping.

RF mapping was completed at the beginning of each experimental session. Each trial began with a fixation spot [a black circle of radius 0.5° dva, degrees of visual angle; color (0.08, 0.08, 0.08), within a scale where red–green–blue values (0, 0, 0) code for black and values (1, 1, 1) code for white] presented at the center of the screen [uniform gray background, color (0.5, 0.5, 0.5)]. After the monkey acquired and maintained fixation for 600 to 800 ms (duration selected randomly on each trial), a series of visual stimuli were presented [one at a time; each stimulus was a white filled square, size 1° × 1° dva; color (1, 1, 1); each stimulus duration was ~100 ms and interstimulus interval was 500 ms; 2 to 8 stimuli per trial; the number of stimuli was selected randomly on each trial]. Stimuli locations were selected randomly from a grid covering the left side of the screen (distance between two adjacent grid locations was ~2° dva; we obtained a minimum of 5 presentations of each location on the grid). Monkeys received a juice reward if they successfully maintained fixation during the trial (intertrial duration was 100 ms). Failures to maintain fixation during the trial were not rewarded and were followed by a 2,000 ms intertrial duration. Manually thresholded neuronal spike counts from each recorded channel were recorded during each trial and were used to prepare online plots of neuronal responses to visual stimuli. The RF of each channel was defined as location(s) with highest neuronal activity. Location with the most overlap between RFs of all recorded channels was used to position stimuli during Look-Avoid tasks.

### Behavioral Tasks: Look and Avoid.

Each behavioral trial began with a fixation spot [a blue circle of radius 0.5° dva; color (0.08, 0.08, 0.78)] presented at the center of the screen [uniform gray background, color (0.5, 0.5, 0.5)]. After the monkey acquired and maintained fixation for 600 to 800 ms (duration selected randomly on each trial), a cue appeared (colored square frame, size 1° × 1° dva) for ~50 ms at a randomly selected location (1 of 4 possible locations separated by 90° polar angle; same eccentricity during a session). Eccentricity from fixation ranged from 5° to 7° dva on different sessions. Cue location was selected randomly on each trial with equal probability, independent of the previous trial. Cue presentation was followed by a delay period. Delay duration was selected randomly on each trial from a set of ranges. On a majority of sessions, the range was 1,600 to 1,800 ms, but it could be as short as 1,000 to 1,200 ms, or as long as 2,000 to 2,200 ms on some sessions, depending on the animal’s motivation. At the end of the delay period, the fixation spot disappeared, and the response targets appeared. Monkeys received a juice reward for making a saccadic eye movement to the correct target and maintaining gaze on the target for 200 ms. Intertrial duration after a correct response was 100 ms. Failures to acquire fixation, breaks of fixation during the trial, or incorrect eye movements were not rewarded and were followed by a 2,000 ms intertrial duration. Such erroneous (nonrewarded) trials were rerun immediately until the monkey made a correct response, and these trial repeats were not used in the analysis.

*Look task.* The cue color indicated the task. The cue color was black [open square, color (0.08, 0.08, 0.08)] for monkey AQ and green [open square, color (0.08, 0.78, 0.08)] for monkey HB. On 93% of trials, after the delay period, two targets appeared [filled blue circles of radius 1° dva, color (0.08, 0.08, 0.78)]. One of the targets always appeared at the previously cued location while the other appeared at a randomly selected one of the other of the 3 remaining locations (location selected randomly on each trial). To be rewarded, monkeys had to make a saccadic eye movement to the target at the cued location. On 7% of trials (probe trials), after the delay, only one target appeared [filled black circle, color (0.08, 0.08, 0.08); at cued or 180° opposite], and monkeys had to make a saccadic eye movement to the probe target to be rewarded.

*Avoid task*. Avoid trials were identical to the Look task, except that to be rewarded monkeys had to make a saccadic eye movement to the novel target—the one not previously cued. The cue color instructed the task (green for monkey AQ and black for monkey HB). As in the Look task, probe trials occurred on 7% of trials.

To prevent well-established task switching costs, Look and Avoid tasks were run in blocks (block duration varied, typically ranging from 150 to 400 trials per block; based on monkey motivation). Each session could begin either with a Look or an Avoid block.

To evoke visual responses in neurons, the screen background was filled with a task-irrelevant texture (Fig. 1*A*) beginning 600 ms before cue onset (0 to 200 ms after the monkey acquired the fixation) (5). The background texture consisted of a dense field of 10,000 uniformly oriented lines (width: 2 pixels, length: 2° dva, color: [0.36, 0.36, 0.36], one orientation per background, selected randomly from 0° to 179° in 30° increments). The background texture was presented on ~5/6 of trials; on ~1/6 of trials no texture was presented, and the background remained uniform gray (probability of no texture background or each texture angle thus was ~1/6). Halfway through the delay, a new texture or no-texture background was presented (selected randomly and independently of the first texture).

### Behavior and Neurophysiological Recording Procedures.

Experiments were controlled by a DELL Precision Tower 3620 desktop computer and implemented in Matlab (MathWorks, Natick, MA, USA) using Psychophysics and Eyelink toolboxes (73, 74). Eye position was recorded with an SR Research EyeLink 1,000 desktop mounted eye tracker for online gaze position tracking (sampling rate 60 Hz) and for offline analysis (sampling rate of 1,000 Hz). Stimuli were presented at a viewing distance of 60 cm, on a VIEWPixx3D display (1,920 × 1,080 pixels, vertical refresh rate of 60 Hz).

Neuronal recordings were obtained with 16- or 32-channel linear array electrodes with contacts spaced 75 or 150 mm apart (U-Probes and V-Probes, Plexon, Inc). Electrodes were lowered into the cortex using a hydraulic microdrive (Narishige International) at angles roughly perpendicular to the cortical surface. The RFs of V4 neurons studied were located within the lower contralateral quadrant within ~4° to 8° eccentricity. Neuronal activity was measured against a local reference, a stainless guide tube, which was close to the electrode contacts. Data were amplified and recorded using the Omniplex system (Plexon Inc., Dallas, TX). Wide-band data filtered only in hardware at 0.5 Hz highpass and 8 kHz lowpass were recorded to disk at 40 kHz. Data in each channel were then extracted using 3 SD as a threshold for extracellular spike and classified as multiunit activity. Neuronal activity was z-score normalized by the mean firing rate for individual units.

Recording sites within area V4 were identified by neuronal responses to visual stimuli (*Behavior Tasks: RF Mapping*) and by assessing RF sizes as a function of stimulus eccentricity and RF location (75). During the Look and Avoid tasks, at least one of the four cue locations was positioned within the RFs of the recorded neurons. To evoke activity in the recorded neurons during the pretrial period, the display background was filled with a task-irrelevant texture.

### Quantification and statistical analysis.

*Eye movement analysis.* Gaze position on each trial was offline drift corrected by using median gaze position from 10 previous trials. Drift correction was based on gaze position from 100 to 10 ms before the cue onset, when stable fixation was maintained. We detected saccades offline using an algorithm based on eye velocity changes (76). We next clustered saccades as ending on one of the three potential locations: 1) fixation, 2) correct response target, and 3) wrong response target. The clustering procedure used support vector machine (SVM) algorithm with a Gaussian kernel (29). Saccades directed to the target or distractor had a latency of at least of 50 ms after the response cue (77). For the microsaccade analysis, we used all saccades that did not break the fixation window during the pretrial period, i.e., saccades with amplitudes less than 1 dva. We removed trials if blinks occurred from 100 ms before cue onset to 200 ms after the time of saccade target onset. Data from each recording were inspected for saccade detection accuracy and data recording noise. For reaction time (RT) analyses, values during probe trials were normalized by the corresponding mean RTs from saccades to the Look and Avoid targets which occurred during the vast majority of trials (93%).

*Statistical tests.* For statistical comparisons of paired means, we drew (with replacement) 10,000 bootstrap samples from the original pair of compared values. We then calculated the difference of these bootstrapped samples and derived two-tailed *P* values from the distribution of these differences. All correlations were computed as Pearson coefficients. Distributions of correlation coefficients, computed either across trials for individual units (Fig. 2*C*) or across a population of units (Fig. 3 *C* and *D*), were compared to each other to a mean of zero using the bootstrap method.

*Decoding of V4 activity.* We analyzed neuronal spike rates measured during the late memory delay period (0 to 300 ms prior to the onset of saccadic targets). We used a SVM algorithm (78) to train classifiers to perform a binary classification of the cue location (cue in-RF or cue opposite) from the population delay activity during each experimental session. The SVM algorithm is based on solving a quadratic programming optimization problem to find a separating hyperplane between the two classes in the given data, so that the distance (i.e., margin) between the samples of the two classes is maximal. We also used the radial basis function kernel (RBF) which projects the given data into a higher dimensional space, thus achieving a better separation of the data and improved decoding accuracy (79). A decoding model was trained on the delay activity of all visual units from each recording session and was evaluated separately using a standard leave-one-out cross-validation procedure where all of the dataset but one trial of data was used for training, and that single trial was used for testing, repeated over all available data points in order to minimize the variance. The session’s classification performance was computed from the average decoding accuracy of all cross-validation conditions. Chance performance level (0.5) was confirmed by shuffling labels in the training dataset.

As an additional analysis, we trained the location decoder on spike data during the earlier period of the delay (−600 to −300 ms prior to the onset of saccadic targets). Decoders trained during that earlier period yielded similar performance to that obtained in the late period. During both the Look and Avoid tasks, mean decoder performance in this earlier period exceeded that of chance (Look: 55.4%, *P* < 0.001; Avoid: 56.1%, *P* < 0.001). In addition, location decoders trained on the Look task and tested on Avoid trials on average performed poorer than chance (median = 45.1%, *P* < 0.08).

### Control and inactivation blocks.

In the inactivation experiment, we collected data during two types of experimental sessions: control and inactivation sessions. Control sessions were intended to establish a behavioral and neural baseline during an experimental session. Inactivation sessions measured the impact of the temporary loss of FEF activity on the behavior and the V4 delay activity. During inactivation sessions, monkeys also first completed a control block of Look-MGS and Avoid tasks (*control*, 300 to 400 trials). Next, after the muscimol infusion (~1 h), they completed postinactivation blocks of Look-MGS and Avoid tasks (*inactivation*, 600 to 1,000 trials).

### FEF inactivation.

Prior to inactivation, we located the FEF based on its neurophysiological characteristics and our ability to evoke saccades with electrical stimulation. Electrical microstimulation consisted of 100-ms trains of biphasic current pulses (0.25 ms, 200 Hz) delivered with a Grass stimulator (S88) and two Grass stimulation isolation units (PSIU-6) (Grass Instruments). The FEF was defined as the region from which saccades could be evoked with currents <50 μA (51). In addition, we used 32-channel linear array electrodes with contacts spaced 75 or 150 mm apart (U-Probes and V-Probes, Plexon, Inc) to map out visual and movement-related response fields to corroborate the metrics of the stimulation-evoked saccades (51). Placement of the memory cue during a given inactivation session was determined by the location of evoked saccades and FEF response fields measured on the previous recording/stimulation session at the same FEF site.

During inactivation sessions, we pharmacologically inactivated the right FEF in both monkeys via infusion of 0.5 to 1 μL of the GABA_a_ agonist muscimol (5 mg/ml), using a custom-made injection system as described and demonstrated previously (57, 58). We typically did not perform experiments on consecutive days to provide sufficient recovery from the previous inactivation. Of 34 control experiments, only 2 were completed on the day following an inactivation. Typical duration of the injection was 20 min plus an additional period (~40 min) to maximize the expected behavioral effects (58, 59).

## Supplementary Material

Appendix 01 (PDF)

## Data Availability

Neural activity and behavioral data have been deposited in Figshare (80).

## References

[1] E. K. Miller, J. D. Cohen, An integrative theory of prefrontal cortex function. Annu. Rev. Neurosci. **24**, 167–202 (2001). 11283309 10.1146/annurev.neuro.24.1.167

[2] B. Noudoost, M. H. Chang, N. A. Steinmetz, T. Moore, Top-down control of visual attention. Curr. Opin. Neurobiol. **20**, 183–190 (2010). 20303256 10.1016/j.conb.2010.02.003 PMC2901796

[3] J. H. Reynolds, L. Chelazzi, Attentional modulation of visual processing. Annu. Rev. Neurosci. **27**, 611–647 (2004). 15217345 10.1146/annurev.neuro.26.041002.131039

[4] J. K. Baruni, B. Lau, C. D. Salzman, Reward expectation differentially modulates attentional behavior and activity in visual area V4. Nat. Neurosci. **18**, 1656–1663 (2015). 26479590 10.1038/nn.4141 PMC4624579

[5] H. Supèr, H. Spekreijse, V. A. Lamme, A neural correlate of working memory in the monkey primary visual cortex. Science **293**, 120–124 (2001). 11441187 10.1126/science.1060496

[6] T. vanKerkoerle, M. W. Self, P. R. Roelfsema, Layer-specificity in the effects of attention and working memory on activity in primary visual cortex. Nat. Commun. **8**, 13804 (2017). 28054544 10.1038/ncomms13804 PMC5227065

[7] J. M. Fuster, *The Prefrontal Cortex* (Raven Press, New York, ed. 3, 1997).

[8] F. A. Mansouri, D. J. Freedman, M. J. Buckley, Emergence of abstract rules in the primate brain. Nat. Rev. Neurosci. **21**, 595–610 (2020), 10.1038/s41583-020-0364-5. 32929262

[9] J. D. Wallis, K. C. Anderson, E. K. Miller, Single neurons in prefrontal cortex encode abstract rules. Nature **411**, 953–956 (2001). 11418860 10.1038/35082081

[10] R. P. Hasegawa, B. W. Peterson, M. E. Goldberg, Prefrontal neurons coding suppression of specific saccades. Neuron **43**, 415–425 (2004). 15294148 10.1016/j.neuron.2004.07.013

[11] D. M. Kaplan, L. H. Snyder, The need for speed: eye-position signal dynamics in the parietal cortex. Neuron **76**, 1048–1051 (2012). 23259941 10.1016/j.neuron.2012.12.005

[12] N. P. Bichot, A. F. Rossi, R. Desimone, Parallel and serial neural mechanisms for visual search in macaque area V4. Science (80-). **308**, 529–534 (2005). 10.1126/science.1109676 15845848

[13] S. J. Luck , Neural mechanisms of spatial selective attention in areas V1, V2, and V4 of macaque visual cortex. J. Neurop **77**, 24–42 (1997). 10.1152/jn.1997.77.1.24 9120566

[14] B. C. Motter, Focal attention produces spatially selective processing in visual cortical areas V1, V2, and V4 in the presence of competing stimuli. J. Neurophysiol. **70**, 909–919 (1993). 8229178 10.1152/jn.1993.70.3.909

[15] A. C. Snyder, B. M. Yu, M. A. Smith, Distinct population codes for attention in the absence and presence of visual stimulation. Nat. Commun. **9**, 4382 (2018). 30348942 10.1038/s41467-018-06754-5 PMC6197235

[16] L. Stănişor, C. van derTogt, C. M. A. Pennartz, P. R. Roelfsema, A unified selection signal for attention and reward in primary visual cortex. Proc. Natl. Acad. Sci. U.S.A. **110**, 9136–9141 (2013). 23676276 10.1073/pnas.1300117110 PMC3670348

[17] D. Mendoza-halliday, S. Torres, J. C. Martinez-trujillo, Sharp emergence of feature-selective sustained activity along the dorsal visual pathway. Nat. Publ. Gr. **17**, 1255–1262 (2014). 10.1038/nn.3785 PMC4978542 25108910

[18] J. M. Fuster, G. E. Alexander, Neuron activity related to short-term memory. Science (80-) **173**, 652–654 (1971). 10.1126/science.173.3997.652 4998337

[19] S. Funahashi, M. V. Chafee, P. S. Goldman-Rakic, Prefrontal neuronal activity in rhesus monkeys performing a delayed anti-saccade task. Nature **365**, 753–756 (1993). 8413653 10.1038/365753a0

[20] T. Pasternak, M. W. Greenlee, Working memory in primate sensory systems. Nat. Rev. Neurosci. **6**, 97–107 (2005). 15654324 10.1038/nrn1603

[21] N. M. Dotson, S. J. Hoffman, B. Goodell, C. M. Gray, Feature-based visual short-term memory is widely distributed and hierarchically organized. Neuron **99**, 215–226.e4 (2018). 29909999 10.1016/j.neuron.2018.05.026 PMC9339219

[22] K. K. Sreenivasan, J. Vytlacil, M. D’Esposito, Distributed and dynamic storage of working memory stimulus information in extrastriate cortex. J. Cogn. Neurosci. **26**, 1141–1153 (2014). 24392897 10.1162/jocn_a_00556 PMC4379324

[23] T. B. Christophel, P. C. Klink, B. Spitzer, P. R. Roelfsema, J.-D. Haynes, The distributed nature of working memory. Trends Cogn. Sci. **21**, 111–124 (2017). 28063661 10.1016/j.tics.2016.12.007

[24] R. Muhammad, J. D. Wallis, E. K. Miller, A comparison of abstract rules in the prefrontal cortex, premotor cortex, inferior temporal cortex, and striatum. J. Cogn. Neurosci. **18**, 974–989 (2006). 16839304 10.1162/jocn.2006.18.6.974

[25] B. Y. Hayden, J. L. Gallant, Working memory and decision processes in visual area v4. Front. Neurosci. **7**, 18 (2013). 23550043 10.3389/fnins.2013.00018 PMC3582211

[26] D. Zaksas, T. Pasternak, Directional signals in the prefrontal cortex and in area MT during a working memory for visual motion task. J. Neurosci. Off. J. Soc. Neurosci. **26**, 11726–11742 (2006). 10.1523/JNEUROSCI.3420-06.2006 PMC6674769 17093094

[27] Y. Merrikhi , Spatial working memory alters the efficac of input to visual cortex. Nat. Commun. **8**, 1–10 (2017). 28447609 10.1038/ncomms15041 PMC5414175

[28] D. Jonikaitis, B. Noudoost, T. Moore, Dissociating the contributions of frontal eye field activity to spatial working memory and motor preparation. J. Neurosci. **43**, 8681–8689 (2023). 37871965 10.1523/JNEUROSCI.1071-23.2023 PMC10727190

[29] D. Jonikaitis, S. Dhawan, H. Deubel, Saccade selection and inhibition: Motor and attentional components. J. Neurophysiol. **121**, 1368–1380 (2019). 30649975 10.1152/jn.00726.2017

[30] M. A. Lebedev, A. Messinger, J. D. Kralik, S. P. Wise, Representation of attended versus remembered locations in prefrontal cortex. PLoS Biol. **2**, e365 (2004). 15510225 10.1371/journal.pbio.0020365 PMC524249

[31] N. Amador, M. Schlag-Rey, J. Schlag, Primate antisaccades. I. Behavioral characteristics. J. Neurophysiol. **80**, 1775–1786 (1998). 9772238 10.1152/jn.1998.80.4.1775

[32] K. Takeda, S. Funahashi, Prefrontal task-related activity representing visual cue location or saccade direction in spatial working memory tasks. J. Neurophysiol. **87**, 567–588 (2002). 11784772 10.1152/jn.00249.2001

[33] R. H. Carpenter, M. L. Williams, Neural computation of log likelihood in control of saccadic eye movements. Nature **377**, 59–62 (1995). 7659161 10.1038/377059a0

[34] M. C. Dorris, D. P. Munoz, Saccadic probability influences motor preparation signals and time to saccadic initiation. J. Neurosci. Off. J. Soc. Neurosci. **18**, 7015–7026 (1998). 10.1523/JNEUROSCI.18-17-07015.1998 PMC6792986 9712670

[35] A. Z. Khan, D. P. Munoz, N. Takahashi, G. Blohm, R. M. McPeek, Effects of a pretarget distractor on saccade reaction times across space and time in monkeys and humans. J. Vis. **16**, 5 (2016). 10.1167/16.7.5 PMC5833323 27148697

[36] D. M. Milstein, M. C. Dorris, The influence of expected value on saccadic preparation. J. Neurosci. Off. J. Soc. Neurosci. **27**, 4810–4818 (2007). 10.1523/JNEUROSCI.0577-07.2007 PMC6672101 17475788

[37] K. M. Armstrong, J. K. Fitzgerald, T. Moore, Changes in visual receptive fields with microstimulation of frontal cortex. Neuron **50**, 791–798 (2006). 16731516 10.1016/j.neuron.2006.05.010

[38] A. Pasupathy, C. E. Connor, Population coding of shape in area V4. Nat. Neurosci. **5**, 1332–1338 (2002). 12426571 10.1038/nn972

[39] H. Tanigawa, H. D. Lu, A. W. Roe, Functional organization for color and orientation in macaque V4. Nat. Neurosci. **13**, 1542–1548 (2010). 21076422 10.1038/nn.2676 PMC3005205

[40] T. Kim, W. Bair, A. Pasupathy, Neural coding for shape and texture in macaque area V4. J. Neurosci. Off. J. Soc. Neurosci. **39**, 4760–4774 (2019). 10.1523/JNEUROSCI.3073-18.2019 PMC6561689 30948478

[41] S. J. Schein, R. Desimone, Spectral properties of V4 neurons in the macaque. J. Neurosci. Off. J. Soc. Neurosci. **10**, 3369–3389 (1990). 10.1523/JNEUROSCI.10-10-03369.1990 PMC6570187 2213146

[42] B. C. Motter, Neural correlates of attentive selection for color or luminance in extrastriate area V4. J. Neurosci. Off. J. Soc. Neurosci. **14**, 2178–2189 (1994). 10.1523/JNEUROSCI.14-04-02178.1994 PMC6577115 8158264

[43] C. J. McAdams, J. H. Maunsell, Effects of attention on orientation-tuning functions of single neurons in macaque cortical area V4. J. Neurosci. **19**, 431–441 (1999). 9870971 10.1523/JNEUROSCI.19-01-00431.1999 PMC6782389

[44] C. E. Connor, D. C. Preddie, J. L. Gallant, D. C. VanEssen, Spatial attention effects in macaque area V4. J. Neurosci. Off. J. Soc. Neurosci. **17**, 3201–3214 (1997). 10.1523/JNEUROSCI.17-09-03201.1997 PMC6573654 9096154

[45] L. H. Snyder, A. P. Batista, R. A. Andersen, Intention-related activity in the posterior parietal cortex: A review. Vision Res. **40**, 1433–1441 (2000). 10788650 10.1016/s0042-6989(00)00052-3

[46] W. F. Asaad, G. Rainer, E. K. Miller, Neural activity in the primate prefrontal cortex during associative learning. Neuron **21**, 1399–1407 (1998). 9883732 10.1016/s0896-6273(00)80658-3

[47] K. M. Armstrong, M. H. Chang, T. Moore, Selection and maintenance of spatial information by frontal eye field neurons. J. Neurosci. **29**, 15621–15629 (2009). 20016076 10.1523/JNEUROSCI.4465-09.2009 PMC3351279

[48] G. B. Stanton, C. J. Bruce, M. E. Goldberg, Topography of projections to posterior cortical areas from the macaque frontal eye fields. J. Comp. Neurol. **353**, 291–305 (1995). 7745137 10.1002/cne.903530210

[49] J. C. Anderson, H. Kennedy, K. A. C. Martin, Pathways of attention: Synaptic relationships of frontal eye field to V4, lateral intraparietal cortex, and area 46 in macaque monkey. J. Neurosci. Off. J. Soc. Neurosci. **31**, 10872–10881 (2011). 10.1523/JNEUROSCI.0622-11.2011 PMC6623081 21795539

[50] P. H. Schiller, S. D. True, J. L. Conway, Deficits in eye movements following frontal eye-field and superior colliculus ablations. J. Neurophysiol. **44**, 1175–1189 (1980). 6778974 10.1152/jn.1980.44.6.1175

[51] C. J. Bruce, M. E. Goldberg, Primate frontal eye fields. I. Single neurons discharging before saccades. J. Neurophysiol. **53**, 603–635 (1985). 3981231 10.1152/jn.1985.53.3.603

[52] J. D. Schall, D. P. Hanes, K. G. Thompson, D. J. King, Saccade target selection in frontal eye field of macaque. I. Visual and premovement activation. J. Neurosci. Off. J Soc. Neurosci. **15**, 6905–6918 (1995). 10.1523/JNEUROSCI.15-10-06905.1995 PMC6577995 7472447

[53] T. Moore, K. M. Armstrong, Selective gating of visual signals by microstimulation of frontal cortex. Nature **421**, 370–373 (2003). 12540901 10.1038/nature01341

[54] L. B. Ekstrom, P. R. Roelfsema, J. T. Arsenault, G. Bonmassar, W. Vanduffel, Bottom-up dependent gating of frontal signals in early visual cortex. Science **321**, 414–417 (2008). 18635806 10.1126/science.1153276 PMC3011100

[55] G. G. Gregoriou, A. F. Rossi, L. G. Ungerleider, R. Desimone, Lesions of prefrontal cortex reduce attentional modulation of neuronal responses and synchrony in V4. Nat. Neurosci. **17**, 1003–1011 (2014). 24929661 10.1038/nn.3742 PMC4122755

[56] J. Hüer, P. Saxena, S. Treue, Pathway-selective optogenetics reveals the functional anatomy of top-down attentional modulation in the macaque visual cortex. Proc. Natl. Acad. Sci. U.S.A. **121**, e2304511121 (2024). 38194453 10.1073/pnas.2304511121 PMC10801865

[57] B. Noudoost, T. Moore, Control of visual cortical signals by prefrontal dopamine. Nature **474**, 372–375 (2011). 21572439 10.1038/nature09995 PMC3117113

[58] B. Noudoost, K. L. Clark, T. Moore, A distinct contribution of the frontal eye field to the visual representation of saccadic targets. J. Neurosci. Off. J. Soc. Neurosci. **34**, 3687–3698 (2014). 10.1523/JNEUROSCI.3824-13.2014 PMC3942584 24599467

[59] E. C. Dias, M. A. Segraves, Muscimol-induced inactivation of monkey frontal eye field: Effects on visually and memory-guided saccades. J. Neurophysiol. **81**, 2191–2214 (1999). 10322059 10.1152/jn.1999.81.5.2191

[60] T. Moore, K. M. Armstrong, M. Fallah, Visuomotor origins of covert spatial attention. Neuron **40**, 671–683 (2003). 14622573 10.1016/s0896-6273(03)00716-5

[61] G. T. Saber, F. Pestilli, C. E. Curtis, Saccade planning evokes topographically specific activity in the dorsal and ventral streams. J. Neurosci. **35**, 245–252 (2015). 25568118 10.1523/JNEUROSCI.1687-14.2015 PMC4287145

[62] C. M. Niell, M. P. Stryker, Modulation of visual responses by behavioral state in mouse visual cortex. Neuron **65**, 472–479 (2010). 20188652 10.1016/j.neuron.2010.01.033 PMC3184003

[63] E. Zagha, A. E. Casale, R. N. S. Sachdev, M. J. McGinley, D. A. McCormick, Motor cortex feedback influences sensory processing by modulating network state. Neuron **79**, 567–578 (2013). 23850595 10.1016/j.neuron.2013.06.008 PMC3742632

[64] M. Vinck, R. Batista-Brito, U. Knoblich, J. A. Cardin, Arousal and locomotion make distinct contributions to cortical activity patterns and visual encoding. Neuron **86**, 740–754 (2015). 25892300 10.1016/j.neuron.2015.03.028 PMC4425590

[65] T. Moore, M. Zirnsak, Neural mechanisms of selective visual attention. Annu. Rev. Psychol. **68**, 47–72 (2017). 28051934 10.1146/annurev-psych-122414-033400

[66] A. D. Milner, M. A. Goodale, The Visual Brain in Action (Oxford University Press, ed. 2, 2006).

[67] A. Mueller, R. M. Krock, S. Shepard, T. Moore, Dopamine receptor expression among local and visual cortex-projecting frontal eye field neurons. Cereb. Cortex **30**, 148–164 (2020). 31038690 10.1093/cercor/bhz078 PMC7029694

[68] A. F. T. Arnsten, Catecholamine influences on dorsolateral prefrontal cortical networks. Biol. Psychiatry **69**, e89–e99 (2011). 21489408 10.1016/j.biopsych.2011.01.027 PMC3145207

[69] S. Funahashi, Prefrontal cortex and working memory processes. Neuroscience **139**, 251–261 (2006). 16325345 10.1016/j.neuroscience.2005.07.003

[70] D. Jonikaitis, S. Zhu, Action space restructures visual working memory in prefrontal cortex. bioRxiv [Preprint] (2023). 10.1101/2023.08.13.553135 (Accessed 13 August 2023).

[71] S. Zhang , Selective attention. Long-range and local circuits for top-down modulation of visual cortex processing. Science **345**, 660–665 (2014). 25104383 10.1126/science.1254126 PMC5776147

[72] B. M. Lawrence, L. H. Snyder, Comparison of effector-specific signals in frontal and parietal cortices. J. Neurophysiol. **96**, 1393–1400 (2006). 16723409 10.1152/jn.01368.2005

[73] D. H. Brainard, The psychophysics toolbox. Spat. Vis. **10**, 433–436 (1997). 9176952

[74] F. W. Cornelissen, E. M. Peters, J. Palmer, The eyelink toolbox: Eye tracking with MATLAB and the psychophysics toolbox. Behav. Res. Methods, Instruments, Comput. **34**, 613–617 (2002). 10.3758/bf03195489 12564564

[75] R. Gattass, A. P. Sousa, C. G. Gross, Visuotopic organization and extent of V3 and V4 of the macaque. J. Neurosci. **8**, 1831–1845 (1988). 3385477 10.1523/JNEUROSCI.08-06-01831.1988 PMC6569322

[76] R. Engbert, R. Kliegl, Microsaccades uncover the orientation of covert attention. Vision Res. **43**, 1035–1045 (2003). 12676246 10.1016/s0042-6989(03)00084-1

[77] B. Fischer, R. A. Boch, Saccadic eye movements after extremely short reaction times in the monkey. Brain Res. **260**, 21–26 (1983). 6402272 10.1016/0006-8993(83)90760-6

[78] R. O. Duda, P. E. Hart, D. G. Stork, Pattern Classification (John Wiley and Sons, Inc., New York, ed. 2, 2001).

[79] C. Cortes, V. Vapnik, Support-vector networks. Mach. Learn. **20**, 273–297 (1995).

[80] D. Jonikaitis, R. Xia, T. Moore, Data from “Robust encoding of stimulus-response mapping in visual cortex.” Figshare. 10.6084/m9.figshare.28344671. Deposited 4 February 2025. PMC11892596 39993188

