# Supplementary material for: Robust encoding of stimulus–response mapping by neurons in visual cortex
Source: Proc Natl Acad Sci U S A. 2025 Feb 24;122(9):e2408079122. doi: 10.1073/pnas.2408079122 (PMC11892596; doi:10.1073/pnas.2408079122)
Supplement: Supplementary file 1 — Appendix 01 (PDF) [file pnas.2408079122.sapp.pdf]

PNAS

Supporting Information for

Robust Encoding of Stimulus-Response Mapping by Neurons in Visual Cortex

Donatas Jonikaitis, Ruobing Xia, and Tirin Moore

Tirin Moore
Email: tirin@stanford.edu

This PDF file includes:

Supporting text
Figures S1 to S3
SI References

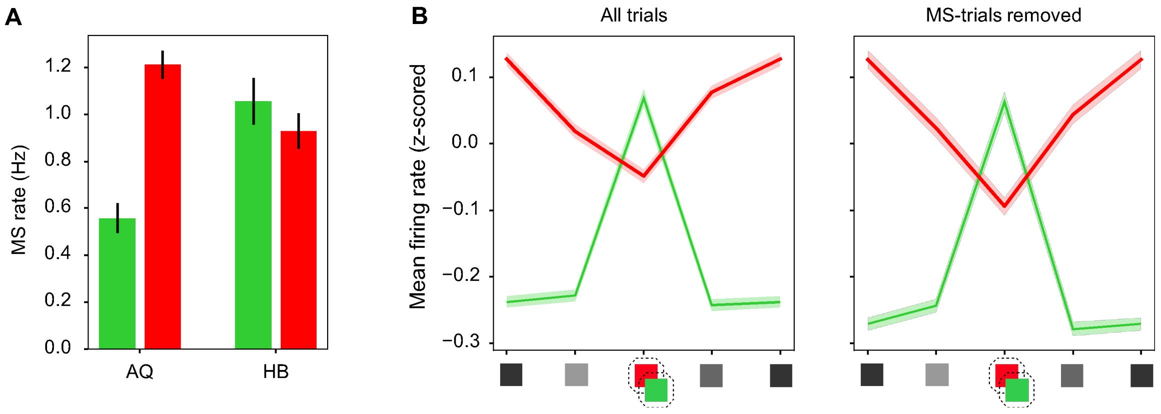

Fig. S1. Microsaccades (MS), also called fixational eye movements, are small-amplitude saccadic eye movements that naturally occur ~1/sec during fixation. They have been shown to affect neuronal activity within visual cortex (Bair &O'Keefe, 1998; Leopold &Logothetis, 1998). In our experiments, we found that both animals made microsaccades during the delay period in both the Look and Avoid tasks (A). The microsaccade rate was similar to what has been reported (AQ: MS rate = 0.79/sec, HB: MS rate=0.99/sec) (Bair & O'keefe 1998; Leopold & Logothetis 1998; Engel et al., 2016). The MS rate varied between the two tasks in one animal (AQ: $p<0.001$, HB: $p=0.33$). To rule out the possible influence of microsaccades on the pattern of delay activity, we removed trials where at least one microsaccade occurred during the last 1 sec of the delay period (14,294 trials were removed out of 31,280 trials in total). In the remaining trials without microsaccades, the pattern of spatial tuning differed between the two tasks in the same way as that in the whole dataset. Specifically, activity was greater in the cue-RF condition during the Look task but was reduced in the Avoid task (B). This control analysis demonstrates that the differed pattern of V4 delay activity between the two tasks is not an effect of microsaccades during the delay period, but rather depends on the stimulus-response mapping rule.

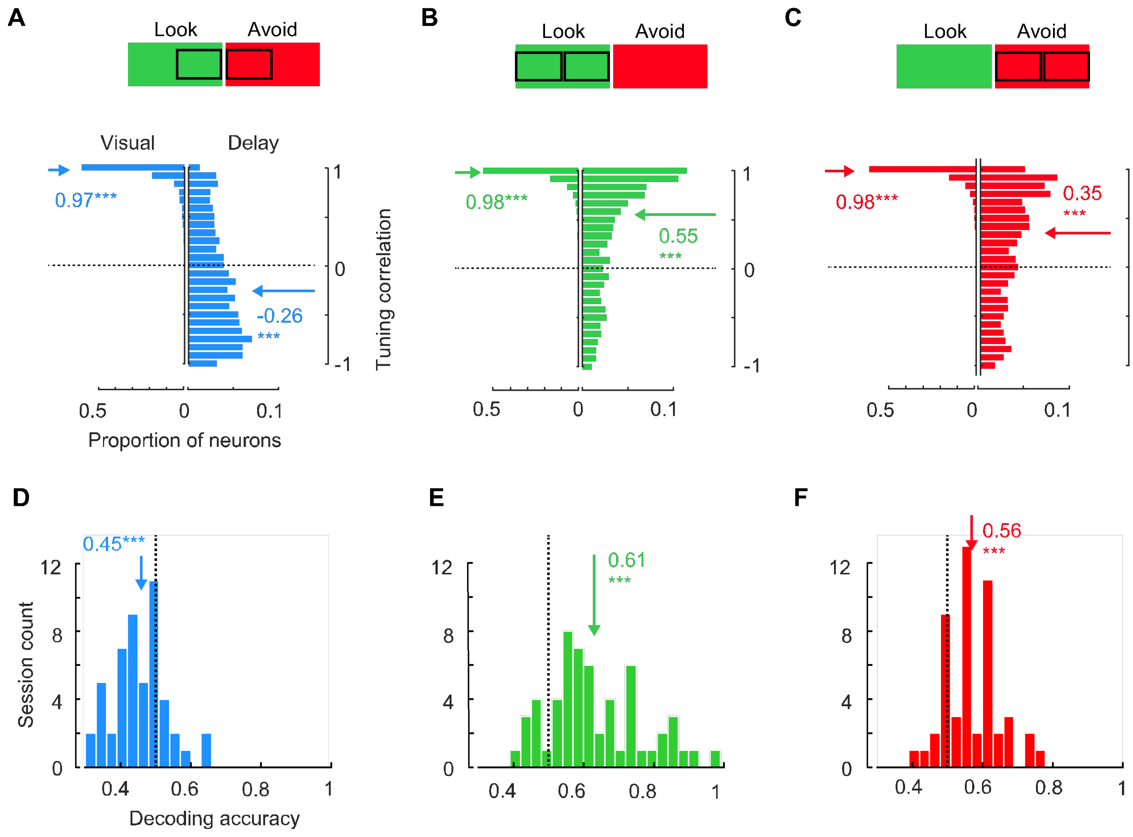

Fig. S2. Given that Look and Avoid tasks were run in blocks (see Methods), we sought to rule out the possibility that the observed difference in response pattern between the two tasks resulted from a time-dependent drift of neuronal activity. To address this, we performed a cross-half-block control analysis in which we divided each block into two halves (first and second) and repeated the cross-condition measures used in the results section. Specifically, for both tuning correlation and cross-task decoding, we examined the results for halves across different blocks in comparison to halves within the same block. When considering halves across blocks, we focused on the second half of the first block (e.g. a Look block) and the first half of the second block (e.g. an Avoid block) (A), which share the same time span as the within block analyses (B-C). In the cross-block analysis, we found that the cue-location tuning for individual units is highly correlated between the Look and Avoid tasks during the visual period, but is negatively correlated during the delay period (A), consistent with our findings in the main results (Figure 3C). By contrast, the tuning functions during the delay period remain positively correlated within the Look (B) and avoid blocks (C). Similarly, cross-block cross-half decoding shows below-chance accuracy (D), as observed in the main result (C), whereas the within-block cross-half decoding shows above-chance predictability in both the Look (E) and Avoid tasks (F). These results indicate that the difference in neural response pattern between the two tasks was not a result of time-dependent drifts, but rather depends on the switch of task rules.

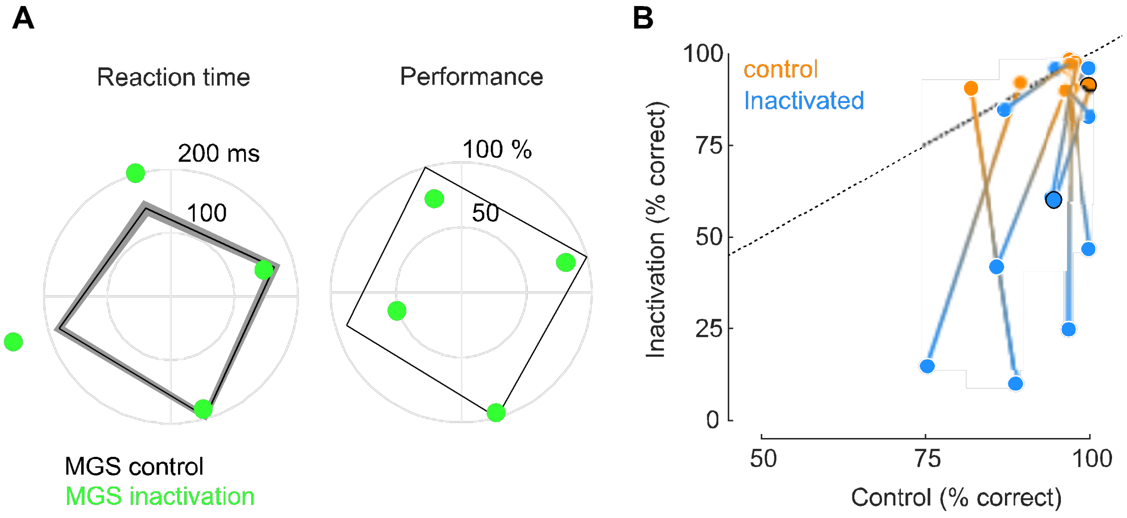

Fig. S2. (A) Behavioral effects of FEF inactivation in an example session. Increased reaction times and response errors observed at MGS target locations within the contralateral visual space following inactivation. (B) Comparison of MGS errors for the control (orange) and contralateral (blue) hemifields in the two monkeys. Lines connect same session data. Dotted line denotes unity. Example session in B is highlighted with black outline.

SI References

1. Bair, W., &O'Keefe, L. P. (1998). The influence of fixational eye movements on the response of neurons in area MT of the macaque. *Visual Neuroscience*, *15*(4), 779–786. https://doi.org/10.1017/s0952523898154160

2. Leopold, D. A., &Logothetis, N. K. (1998). Microsaccades differentially modulate neural activity in the striate and extrastriate visual cortex. *Experimental Brain Research*, *123*(3), 341–345. https://doi.org/10.1007/s002210050577
